# A Bright IDEA

**DOI:** 10.15252/msb.20209502

**Published:** 2020-04-06

**Authors:** Christopher Jackson, David Gresham

**Affiliations:** ^1^ Center for Genomics and Systems Biology Department of Biology New York University New York NY USA

**Keywords:** Chromatin, Epigenetics, Genomics & Functional Genomics, Computational Biology, Methods & Resources

## Abstract

Transcription factors (TFs) control the rate of mRNA production. Technological advances have made the task of measuring mRNA levels for all genes straightforward, but identifying causal relationships between TFs and their target genes remains an unsolved problem in biology. In their recent study, McIsaac and colleagues (Hackett *et al*, 2020) apply a method for inducing the overexpression of a TF and studying the dynamics with which all transcripts respond. Using time series analysis, they are able to resolve direct effects of TFs from secondary effects. This new experimental and analytical approach provides an efficient means of defining gene regulatory relationships for all TFs.

## Graphical abstract

Identifying causal regulatory relationships between transcription factors(TFs) and their target genes remains an unsolved problem. In their recent study, McIsaac and colleagues develop a method to resolve direct effects of TFs from secondary effects.

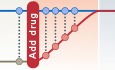

Defining a functional map of the cell that connects regulators with their targets is essential for understanding, and engineering, cellular behavior (Chasman *et al*, 2016). The budding yeast *Saccharomyces cerevisiae* is an ideal organism for defining and studying gene regulatory networks (Hughes & de Boer, 2013) using global methods from DNA microarrays (Hughes *et al*, 2000) to single‐cell RNA sequencing (Jackson *et al*, 2020).

One of the biggest challenges to figuring out the overall transcriptional regulatory network has been in designing experimental assays that provide unambiguous information. Typically, experimental approaches start from some initial steady state; the organism is then perturbed in some way after which it adapts to the perturbation and enters a new steady state (Fig 1A). To study the role of a specific TF in mediating the observed response, two general designs are used. One type of experiment targets a specific TF, knocking it down or out, and then measuring the effect on gene expression. With this experiment, you can measure how the organism has acclimatized to loss of the TF (Fig 1B). The second design treats the entire organism with some chemical or environmental stressor and measures what happens as a dynamic response (Fig 1C). The best‐designed experiments for identifying TF‐mediated gene expression regulation combine both genetic changes and an environmental signal, measuring the response over time in both unaltered and altered genetic backgrounds (Fig 1D). This provides information on what is changing and how the changes are being effected. However, this approach is more difficult experimentally as it takes considerable resources to undertake all the gene expression measurements, and in many cases, the targeted genetic change does not have an effect.

**Figure 1:**
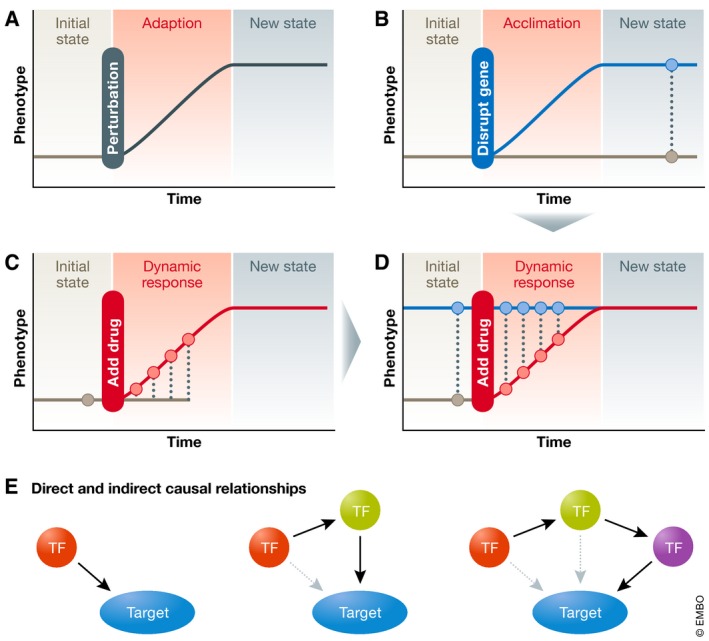
Experimental design for network inference (A) Experimental designs to generate data used in gene network reconstruction use a variety of perturbations including (B) genetic, (C) environmental (e.g., drug or nutrients), or (D) their combination enabling the identification of genotype specific responses (e.g., wild type in gray versus mutant in blue). (E) Transcription factors may regulate target genes directly or indirectly by influencing the activity of intermediate transcription factors.

A second big challenge to defining a global gene regulatory network is that even well‐designed, expensive experiments have difficulty establishing causality. If a TF is disrupted (using, for example, CRISPR/Cas9‐induced loss of function), then a change to a target gene may be due to a direct causal relationship or due to an indirect effect that requires the involvement of other intermediate TFs (Fig 1E). Often, concluding that there is a direct causal relationship between the TF and gene requires additional evidence of a TF–gene relationship, such as physical localization using chromatin immunoprecipitation (ChIP).

In their recent work using yeast, Hackett *et al* (2020) present IDEA (the Induction Dynamics gene Expression Atlas), an organism‐scale transcriptional regulatory network. The authors built this network by engineering 200 yeast strains so that each would rapidly induce the expression of a single TF in response to the chemical signal β‐estradiol (McIsaac *et al*, 2013). Each strain was grown separately in a chemostat and then treated with the inducer, followed by gene expression measurements multiple times over the following hour and a half. Using this approach allows identification of the immediate effect of increasing the expression of a specific transcription factor on the dynamics of the global transcriptional response. In total, the IDEA data set contains information on overexpression of 200 individual TFs using more than 1,600 genome‐wide expression measurements.

The experimental design used by Hackett *et al*, comprising precise induction and time series analysis, is elegantly conceived for learning gene regulatory networks. The combination of time series data with individual TF perturbations allows Hackett *et al* to distinguish direct from indirect relationships between TFs and their target genes. For each TF overexpression time series, the authors fit sigmoidal “impulse” models for every gene. When genes respond quickly to a TF, it is more likely that the relationship is direct; when they respond more slowly, an indirect relationship that includes intermediate regulation is more likely. This is a clever way to identify direct causality without requiring physical localization experiments like ChIP‐seq.

Using these data, the authors constructed a global gene regulatory network that has high predictive value. This network was then validated by predicting the targets that would be affected by overexpression of several regulatory genes that were not in their original screen. Many (but not all) predictions are validated experimentally, demonstrating the utility of their approach.

Understanding how genes are regulated is one of the core problems in biology. Complex genetic diseases and dysregulated cellular states that result from tumorigenesis are inherently network problems. Bioengineering and synthetic biology successes have almost exclusively introduced new and heterologous regulatory elements during design, instead of integrating with the natural network. Having a complete and predictive gene regulatory network, even of a simple eukaryote, will be a major breakthrough. The experimental and modeling advances, presented by Hackett *et al*, bring us closer to realizing this goal.
